# The Small Molecule Compound Eupalinolide B Ameliorates Depressive Behaviors and Neuropathic Pain in Mice With Spared Nerve Injury: Integrating Network Pharmacology, Molecular Docking, Bioinformatics, Molecular Dynamics Simulation and Experimental Verification

**DOI:** 10.1002/cns.70872

**Published:** 2026-04-10

**Authors:** Xuesong Yang, Fan Jiang, Yanqiong Wu, Kun Chen, Hongbing Xiang

**Affiliations:** ^1^ Department of Anesthesiology and Pain Medicine, Hubei Key Laboratory of Geriatric Anesthesia and Perioperative Brain Health, Wuhan Clinical Research Center for Geriatric Anesthesia, Tongji Hospital, Tongji Medical College Huazhong University of Science and Technology Wuhan China; ^2^ Institute of Anesthesiology & Pain (IAP), Department of Anesthesiology, Taihe Hospital Hubei University of Medicine Shiyan China; ^3^ Department of Anesthesiology Wenchang People's Hospital Wenchang China

**Keywords:** bioinformatics, depression, Eupalinolide B, network pharmacology, neuropathic pain, synaptic plasticity

## Abstract

**Background:**

Neuropathic pain (NP) frequently co‐occurs with depression (DP), exhibiting complex pathogenesis and limited clinical treatment options. This study aims to investigate the efficacy of Eupalinolide B (EB) in alleviating NP co‐occurring with DP and its potential molecular mechanisms.

**Methods:**

Combining network pharmacology, molecular docking, and molecular dynamics simulations to screen potential targets for EB, validated through transcriptomic data. Using a sciatic nerve branch‐preserving injury (SNI) mouse model, we assessed pain and depression‐like behaviors through von Frey testing, hot plate testing, tail suspension testing, forced swimming testing, and open field testing. Concurrently, Western blotting, immunofluorescence, and Nissl staining were employed to analyze relevant molecules and neuropathological alterations.

**Results:**

Network pharmacology and bioinformatics analysis identified EGFR, PTGS2, and JUN as the key targets for EB in treating NP combined with DP. Behavioral studies showed that 20 mg/kg of EB significantly alleviated pain in SNI mice and improved depressive‐like behaviors. Mechanism research indicated that EB downregulated the expression of EGFR and PTGS2, inhibited the activation of microglia and astrocytes, and reduced neuronal damage. Additionally, EB could upregulate the expression of synaptic proteins (PSD95, SYN1, and BDNF) in the hippocampus.

**Conclusion:**

EB alleviates neuroinflammation by reducing EGFR and PTGS2 protein expression, modulates synaptic plasticity, and improves pain‐depression comorbidity. EB may represent a promising therapeutic approach for pain‐related depression.

## Graphical abstract

Through network pharmacology, molecular docking, molecular dynamics simulations, and bioinformatics techniques, three potential core targets of EB for the treatment of pain‐depression comorbidity (EGFR, PTGS2, and JUN) were identified. Subsequent animal studies demonstrated that EB alleviates pain and depression‐like behaviors by inhibiting glial cell activation, reducing neuronal injury, and restoring synaptic plasticity.

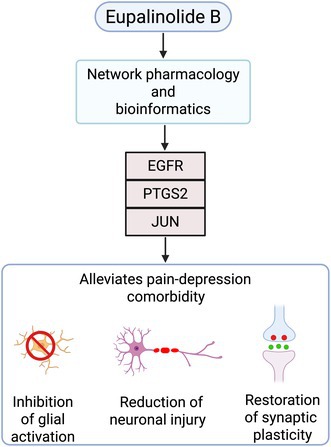

## 1 Introduction

Neuropathic Pain (NP) is usually caused by damage or abnormal function of the somatosensory nervous system. Patients often present with symptoms such as spontaneous pain, abnormal nociception, and nociceptive sensitivity, and lesions can involve the central or peripheral nervous system [1]. Epidemiologic data indicate that the prevalence of NP is approximately 7%–10% in the general population, with a higher prevalence in females and older age groups [2]. Common pain sites include the low back, lower extremities, neck, and shoulder regions, significantly affecting patients' quality of life. However, current conventional analgesic treatments are only 30%–40% effective [3]. There is a strong association between chronic pain and mood disorders, with depression enhancing pain perception and persistent pain triggering depression in a vicious circle. Pain‐Depression Dyad (PDD) is a relatively common chronic pathological condition in clinical practice, mainly characterized by the coexistence of NP and depressive symptoms. According to the definition of the International Association for the Study of Pain, pain is not only a sensory experience, but is also influenced by multiple emotional, psychological and social factors [4, 5]. In recent years, research has increasingly focused on the mechanisms associated with NP and depressive behaviors, as well as on advances in related therapeutic strategies [6, 7, 8].

Spared Nerve Injury (SNI) is a classic animal model of neuropathic pain that mimics the pathology and behavioral characteristics of chronic neuropathic pain in humans by selectively preserving a branch of the sciatic nerve and severing the remaining nerve branches [9, 10]. This model not only induces persistent pain behaviors in mice but also leads to neuropsychiatric disorders, including anxiety and depressive behaviors [11, 12]. It has been reported that mice with SNI‐induced neuropathic pain exhibit anxiety‐depressive behaviors 6 weeks after SNI surgery [13, 14]. In addition, Zou et al. demonstrated a decrease in norepinephrine levels in the hippocampus of a mouse model of PDD, an increase in microglial cell activation, and an increase in proinflammatory cytokines, such as TNF‐α and IL‐1β, after SNI, suggesting that inflammation and oxidative stress may be involved in the pathogenesis of PDD [15].

Eupalinolide B (EB) is a sesquiterpene lactone compound derived from Eupatorium species, known for its diverse pharmacological properties, particularly its anti‐inflammatory and antitumor activities [16, 17, 18]. Zeng et al. have demonstrated that EB functions as a small‐molecule chemotactic agent targeting the deubiquitinase USP7, thereby promoting the ubiquitination‐dependent degradation of Keap1 [19]. This process enhances Nrf2‐mediated transcription of anti‐neuroinflammatory genes and inhibits microglial overactivation. In a periodontitis model, EB was shown to suppress disease progression by targeting the ubiquitin‐conjugating enzyme UBE2D3 [20]. Moreover, EB significantly alleviated acute lung injury induced by lipopolysaccharide (LPS) in mice [17], and in rheumatoid arthritis, it promoted apoptosis and autophagy by modulating the AMPK/mTOR/ULK‐1 signaling pathway, thereby reducing inflammatory symptoms [21]. These findings suggest that EB may have therapeutic potential in managing NP and associated depressive symptoms. However, its precise role and mechanisms in comorbid NP and DP models remain largely unclear and warrant further investigation. Therefore, in this study, our aim was to verify the following hypothesis: The small molecule compound EB alleviates NP and related depressive‐like behaviors by regulating neuroinflammation and synaptic plasticity.

Over the past few years, bioinformatics has become a powerful tool for therapeutic medical research, and it plays a key role in revealing complex disease mechanisms and assisting drug screening. Through the in‐depth mining of large‐scale biological data, bioinformatics methods can accurately identify relevant pathways and core targets, providing a scientific basis for disease intervention [22]. Unlike previous studies, this research integrates multiple methodologies, including network pharmacology analysis, bioinformatics validation, molecular docking, molecular dynamics simulations, and animal experimental validation. It aims to systematically screen and identify potential key targets of EB and their underlying molecular mechanisms.

## 2 Materials and Methods

### 2.1 Target Screening for NP and DP

GeneCards, DisGeNET, Online Mendelian Inheritance in Man (OMIM), Comparative Toxicogenomics Database (CTD), and Therapeutic Target Screening (TTS) were used to screen the targets of NP and DP with the keywords “Neuropathic Pain (NP)” and “Depression (DP)”. All targets retrieved from GeneCards, CTD, OMIM, and TTD were collected. For DisGeNET, we collected targets with scoreGDA ≥ 0.4. After integrating the targets extracted from each database and removing duplicates, we obtained the potential associated targets for NP and DP separately. Subsequently, the intersection method was used to identify targets common to both diseases.

### 2.2 Target Screening of EB

The ISMILES coding information of EB was obtained from the PubChem database (https://pubchem.ncbi.nlm.nih.gov/) and entered into the SwissTargetPrediction platform (http://www.swisstargetprediction.ch/) for potential target prediction. Based on the prediction results, the entries with a probability greater than 0 were filtered as alternative targets. In addition, predicted targets for EB were also obtained from the PharmMapper database (https://www.lilab‐ecust.cn/pharmmapper/index.html). After integrating the results from the two databases, duplicates were removed and the potential targets of EB were identified by intersection solving.

### 2.3 Core Targets for Gene Ontology (GO) and Kyoto Encyclopedia of Genes and Genomes (KEGG) Pathway Enrichment Analysis

Core targets were analyzed for GO and KEGG pathway enrichment using the WebGestalt database (http://www.webgestalt.org/). First, the candidate targets were intersected by the online Venn tool (https://bioinfogp.cnb.csic.es/tools/venny/) and imported into the WebGestalt platform. In the analysis, “human ( *Homo sapiens* )” was selected as the species, and the gene functions were annotated and predicted from the three dimensions of molecular function (MF), biological process (BP), and cellular component (CC), respectively. Meanwhile, KEGG pathway enrichment analysis was used to identify relevant biological pathways in order to understand the functions and roles of target genes in systems biology. All enrichment analysis results were visualized by the ggplot2 package in R language. The significance screening thresholds were set at *p* < 0.05 and false positive detection rate (FDR) < 0.05. The top 10 GO items and the top 10 KEGG pathways with the highest enrichment were finally selected for graphical presentation.

### 2.4 Core Target Discovery Based on Protein–Protein Interaction (PPI) Network Analysis

The intersection targets of EB, NP, and DP were screened using the online Venn diagram tool, and a protein–protein interaction network was constructed in the STRING 12.0 database (https://cn.string‐db.org/) with the lowest interaction confidence level set to 0.4. Then, Cytoscape 3.9.1 software was used to visualize and analyze the network topology, focusing on calculating the Degree, Betweenness, and Closeness of the nodes to identify potential key nodes within the network topology. In the visualization process, the Degree value of a node indicates its importance in the network; the warmer the color, the higher the centrality of the protein in the interaction network, which may play a more critical role in biological functions.

### 2.5 Molecular Docking Simulation

First, the 3D structures of target proteins corresponding to candidate genes were obtained from the UniProt protein database (https://www.uniprot.org/). The ligand structure of the small molecule compound EB was downloaded from the PubChem database (https://pubchem.ncbi.nlm.nih.gov/) and imported into ChemBio3D 15.0 software, where it underwent energy minimization using the MM2 force field. The optimized small molecule structure was saved in mol2 format. Subsequently, water molecules were removed and polar hydrogen atoms were added to all protein files, completing the preprocessing of proteins and ligands. A grid box was set to cover each protein domain while ensuring sufficient space for the small molecule to move freely. The docking pocket was defined as a 30 Å × 30 Å × 30 Å cube with a lattice spacing of 0.05 nm. Semi‐rigid molecular docking was performed using AutoDockTools 1.5.7. To ensure docking accuracy, the number of docking runs was set to 10. An average binding energy < −7.0 kcal/mol is generally considered indicative of strong binding affinity between the target protein and compound. The optimal conformation of the small molecule with the lowest binding energy was saved as the final docking result. Next, the docked complexes were structurally visualized using PyMOL 3.1.4.1 software and the binding energy data were plotted as heat maps using the pheatmap package in R language. Further, the top 10 proteins in terms of Degree value were screened by PPI network analysis and intersected with the top 10 targets with the lowest average docking binding energy, and the key targets with the closest interaction with EB and NP were identified by Venn diagrams, which provided the basis for the subsequent experimental validation.

### 2.6 Acquisition and Target Validation of a Neuropathic Pain and Depression Dataset

Neuropathic pain and depression related datasets were obtained from the Gene Expression Omnibus database (GEO: https://www.ncbi.nlm.nih.gov/geo/). The datasets related to depression include GSE81672 and GSE146845. The sequencing data are from the cortex of C57BL/6J mice, including 9 control samples and 6 depression samples. We filtered the raw data using Fastp, removing data with base quality lower than 36 and length less than 20. Then, we used Hisat2 and FeatureCounts and used GRCm39 as the reference file for alignment, ultimately obtaining the transcriptome expression matrix of the depression data. The dataset related to neuropathic pain is GSE24982. The sequencing data are from the DRGs of adult rats L4 and L5, including 10 Sham group samples and 10 neuropathic pain samples. Differential expression analyses were performed on the above datasets using the “limma” R package, with the screening criteria set as |log2(FC)| > 0.5 and *p* < 0.05. Differential expression results were visualized by volcano maps and heat maps drawn by “ggplot2” and “pheatmap” R packages, respectively. Subsequently, the changes in the expression levels of the three core targets (JUN, EGFR and PTGS2) in the Neuropathic Pain and Depression dataset were analyzed to validate their potential disease relevance.

### 2.7 Validation of EB Core Targets and Molecular Dynamics (MD) Simulations

To further validate the binding stability of EB and NP with core targets related to DP, molecular dynamics (MD) simulations were performed. The lowest‐energy binding conformations from molecular docking were selected as the initial protein–ligand complexes. Simulations were carried out using GROMACS 2024.1 with the AMBER ff14SB force field. Ligand topology files were generated using the Antechamber tool in AmberTools 22, based on the GAFF force field, and partial charges were assigned using the AM1‐BCC method. The simulation system was constructed in a cubic water box with a side length of 10 nm, placing the protein–ligand complex at the center and ensuring a minimum distance of 2.0 nm between the protein and the box edge. Solvation was performed using the TIP3P water model, and 0.15 M NaCl was added to neutralize the system. Energy minimization was conducted using the steepest descent algorithm until the maximum force was below 10 kJ/mol/nm. Subsequently, the system underwent a 100 ps NVT equilibration at 303.15 K (using the V‐rescale thermostat with a 1 fs time step), followed by a 100 ps NPT equilibration (using the Parrinello‐Rahman barostat with a target pressure of 1 atm and a 2 fs time step). During equilibration, positional restraints with a force constant of 1000 kJ/mol·nm^2^ were applied to the ligand. After system preparation, a 100 ns production simulation was performed under unrestrained conditions. Periodic boundary conditions (PBC) were applied throughout the simulation, and long‐range electrostatic interactions were treated using the Particle Mesh Ewald (PME) method. Trajectory data were analyzed to evaluate the structural stability and dynamic behavior of the protein–ligand complexes, including root mean square deviation (RMSD), root mean square fluctuation (RMSF), radius of gyration (RoG), and solvent‐accessible surface area (SASA). Binding free energies were calculated using the gmx_MMPBSA v1.5.0 tool based on the trajectory from 90 to 100 ns to assess the binding affinity of the complexes.

### 2.8 Animal

Male C57BL/6J mice (8–10 weeks old) were obtained from the Institute of Laboratory Animal Science, Hubei University of Medicine. Animals were maintained under standardized conditions (22°C–25°C, 45%–65% relative humidity, 12 h light/dark cycle) with free access to food and water.

### 2.9 Drug Administration

EB, a small molecule compound, was sourced from PUSH Bio‐Technology (Chengdu, China). Previous studies have shown that the small molecule compound EB can cross the blood–brain barrier and reduce neuroinflammation by inhibiting microglial activation. Therefore, intraperitoneal injection was used as the route of administration for EB in this study [19]. Dissolve the drug in DMSO, dilute with saline, and administer a single intraperitoneal injection daily from days 23 to 38 post‐SNI modeling.

### 2.10 Neuropathic Pain Models and Behavioral Experiments

SNI modeling surgery procedures and behavioral tests for pain and depression were conducted according to the methods described in previous studies [23, 24]. Detailed methods are shown in Supporting Information S1.

### 2.11 Western Blotting Analysis, Nissl Staining and Immunofluorescence Staining

Detailed methods for this section are shown in Supporting Information S1.

### 2.12 Statistical Analysis

Data are presented as mean ± SD and analyzed using GraphPad Prism v9.0. Normality of data distribution was assessed using the Shapiro–Wilk test. Pain behavioral data were analyzed using two‐way analysis of variance (ANOVA), followed by Bonferroni post hoc tests. Depression‐related behavioral data, immunofluorescence, and Western blot data were analyzed using one‐way analysis of variance (ANOVA), followed by the Bonferroni post hoc test. *p* < 0.05 was considered statistically significant (*p < 0.05, **p < 0.01).

## 3 Results

### 3.1 Screening of EB, NP and DP Related Targets and Cross‐Targets

**FIGURE 1:**
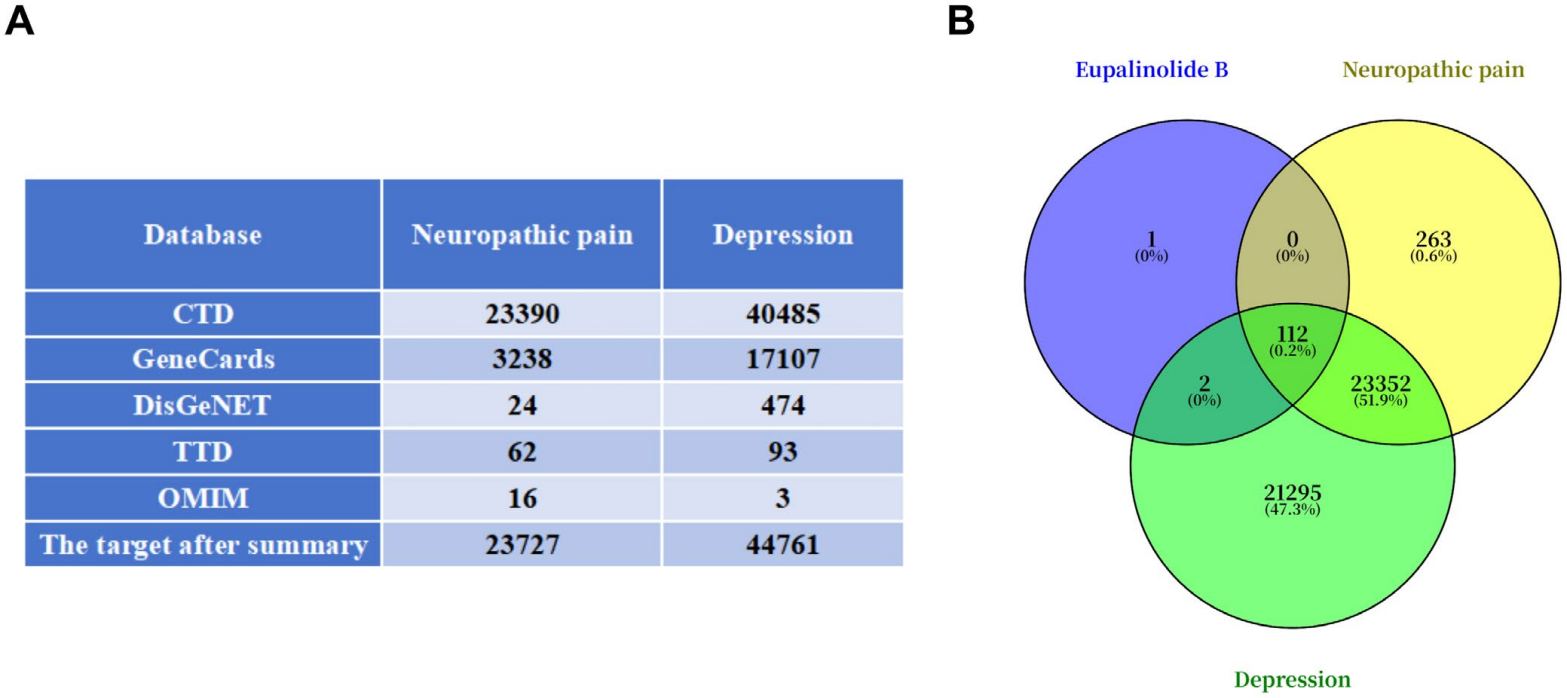
Acquisition of EB, NP, and DP core targets. (A) Disease target information tables were obtained from each database. (B) Venn diagram of intersection targets of EB, NP, and DP.

Thirty‐five EB potential targets with a probability greater than 0 were predicted from the SwissTargetPrediction database. Meanwhile, 85 EB predicted targets were screened from the PharmMapper database. After de‐weighting, a total of 115 EB potential targets were obtained. As shown in Figure 1A, 23,727 neuropathic pain disease targets and 44,761 depression targets were obtained, respectively. The intersection of EB and pain‐depression comorbidity targets was obtained by constructing a Venn diagram, and 112 intersecting targets were obtained, as shown in Figure 1B.

### 3.2 GO and KEGG Enrichment Analysis

**FIGURE 2:**
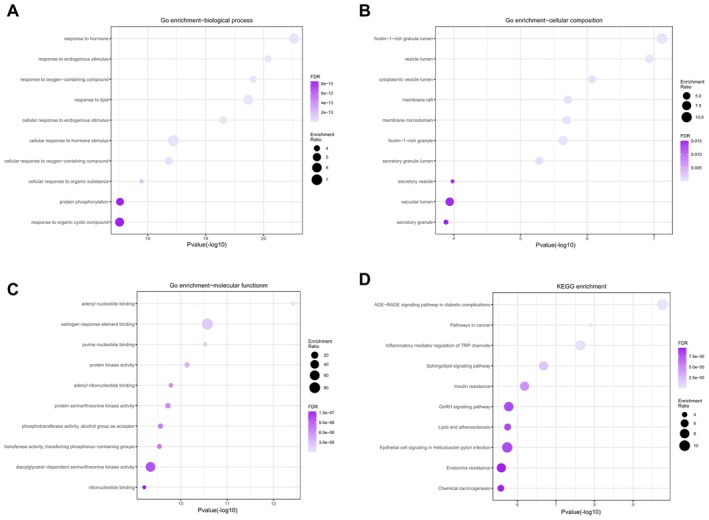
GO, KEGG, and pathway analysis results for 112 intersecting targets. (A) Enriched gene ontology terms for BPs associated with intersecting targets. (B) Enriched gene ontology terms for CCs associated with intersecting targets. (C) Enriched gene ontology terms for MFs associated with intersecting targets. (D) Enriched KEGG pathways for intersecting targets.

GO and KEGG enrichment analyses were performed on the 112 intersecting targets of EB and pain‐depression comorbidity. The top 10 pathways with the smallest *p* values in each section were visualized using a bubble chart (Figure 2). GO analysis indicated that the mechanisms by which EB alleviates neuropathic pain and depression may be closely associated with neural signaling, inflammatory response regulation, and cellular metabolism. Specifically, in biological processes (BP), enriched pathways such as response to endogenous stimulus and response to oxygen‐containing compound suggest EB may alleviate pain and depression by modulating neuronal responses to external stimuli. Regarding cellular components (CC), enriched pathways like secretory granule and cytoplasmic vesicle lumen suggest EB may influence neurotransmitter release and synaptic plasticity by regulating intracellular transport and secretory functions. Regarding molecular functions (MF), enriched pathways like protein kinase activity and adenyl nucleotide binding suggest EB may enhance neurotransmitter synthesis, transport, and receptor binding by regulating signal transduction and enzyme activity. KEGG analysis further reveals potential mechanisms by which EB alleviates neuropathic pain and depression, particularly through regulating key pathways such as inflammatory mediator regulation of TRP channels and sphingolipid signaling pathway. These pathways are associated with neuroinflammation, neurotransmitter imbalance, and pain amplification, suggesting EB may exert synergistic therapeutic effects by exerting anti‐inflammatory actions, modulating neurotransmission, and repairing nervous system damage.

### 3.3 PPI Network Construction and Key Target Discovery

**FIGURE 3:**
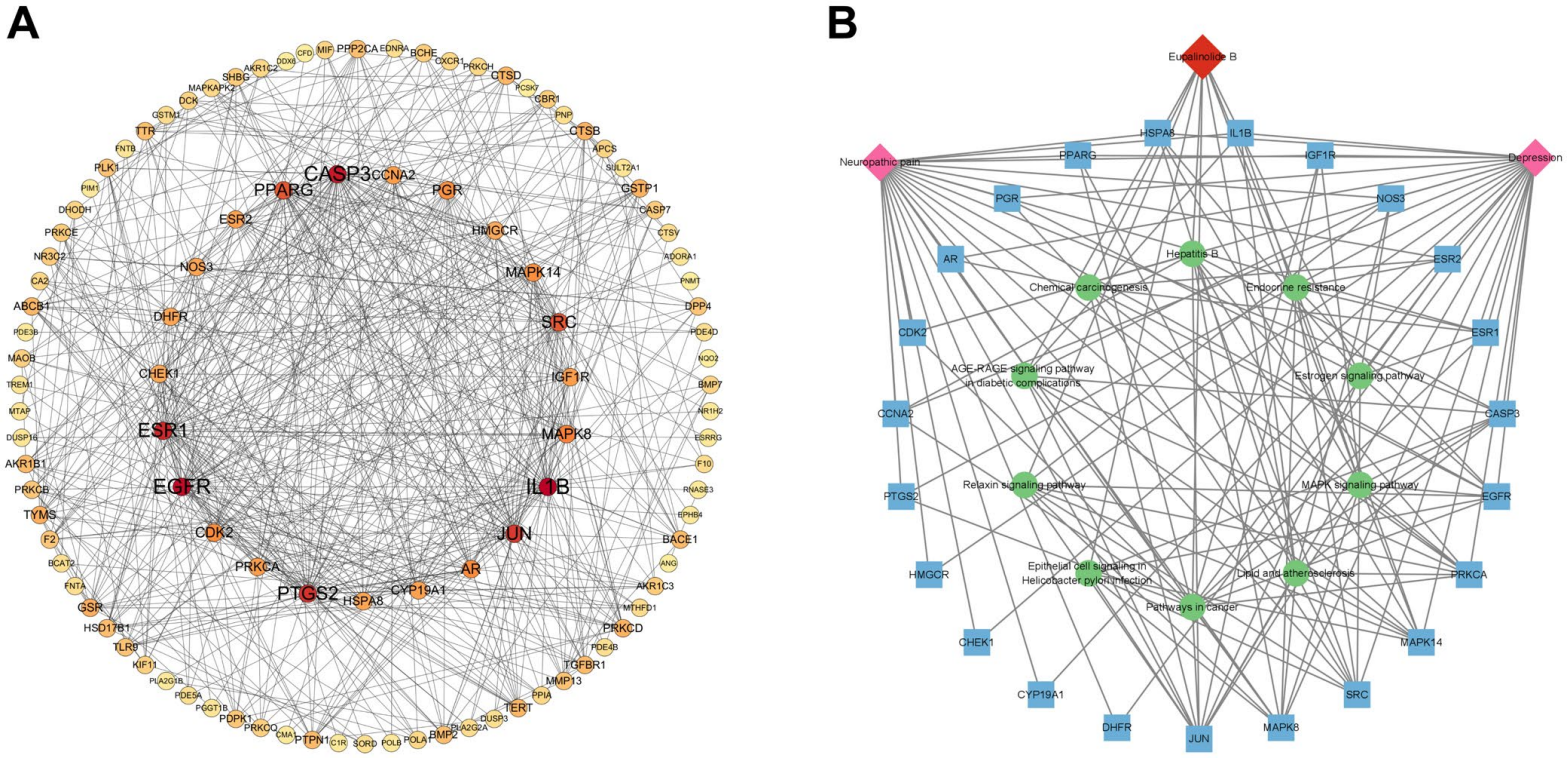
Construction of protein–protein interaction (PPI) network diagram. (A) PPI network and key targets of intersection targets of EB, NP, and DP. (B) Gene‐disease‐drug‐pathway network diagram of 23 core targets.

All 112 targets in the EB therapeutic NP and DP gene sets were imported into the STRING database to construct the PPI network. In order to more clearly reflect the regulatory role of the core targets in the PPI network, the PPI network was visualized by Cytoscape software, and a beautified PPI network graph was obtained (Figure 3A). There are 105 nodes and 596 node edges, and the average node degree value is 11.35. Higher degree values may be core targets, which play a key regulatory role in the PPI network. Twenty‐three targets with degree values ≥ 17 were predicted as core targets. We used core targets to create a disease‐gene‐drug‐target pathway map (Figure 3B).

### 3.4 Molecular Docking Validation

**FIGURE 4:**
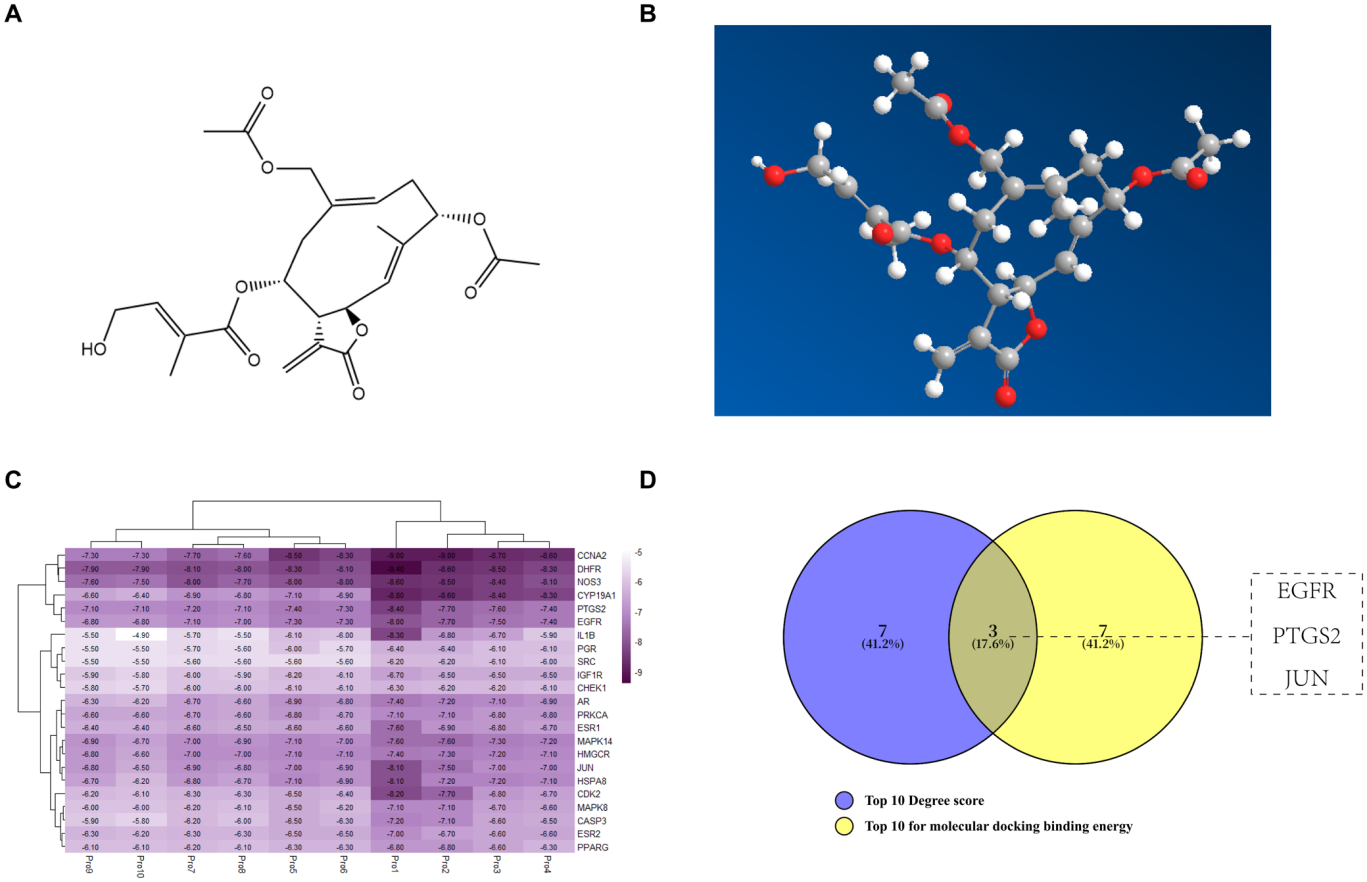
Molecular docking simulation was used to find hub genes. (A) 2D structure diagram of EB. (B) 3D structure of EB. (C) Heat map of binding energy results from 10 molecular dockings of 23 core targets with EB. (D) Schematic representation of Venn diagram intersection results of the target with Degree value top 10 and the top 10 target with the smallest average binding energy of molecular docking.

**FIGURE 5:**
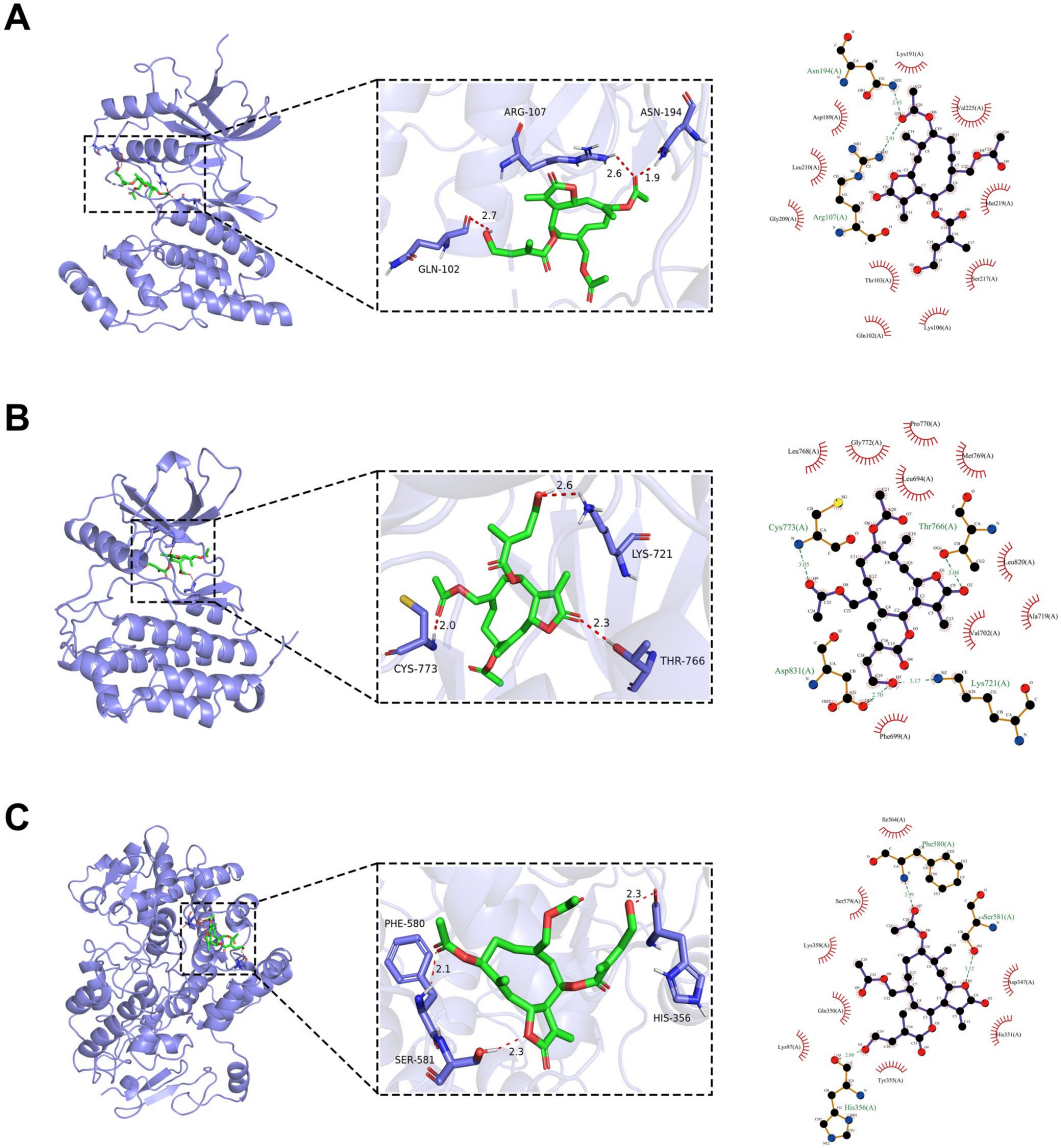
Schematic diagram of the results of molecular docking simulation between EB and three core targets. (A) Schematic diagram of the molecular docking simulation results of EB and JUN protein. (B) Schematic diagram of the molecular docking simulation results of EB and EGFR protein. (C) Schematic diagram of the molecular docking simulation results of EB and PTGS2 protein.

To further evaluate the binding ability between EBs and candidate core targets and their potential interaction modes, we performed molecular docking simulation analysis. The 2D structure of the first EB was obtained from the PubChem database as shown in Figure 4A and imported into ChemBio3D 15.0 software to generate the 3D structure for molecular docking, as shown in Figure 4B. Subsequently, 10 molecular dockings with EB were performed for each of the 23 core candidate targets, and their average binding energies were calculated and organized, and heatmaps were plotted for ranking (Figure 4C). The results showed that EB bound most tightly to 10 targets (Average binding energy < −7 kcal/mol), including DHFR, CCNA2, NOS3, CYP19A1, PTGS2, EGFR, MAPK14, HMGCR, JUN, and HSPA8. The top 10 targets in terms of average binding energy were further analyzed by intersection analysis with the targets with the top 10 Degree values in Table 1, and three common core targets, JUN, EGFR, and PTGS2, were obtained by plotting Venn diagrams (Figure 4D), and the docking patterns were plotted (Figure 5A–C).

**TABLE 1:** The 23 core targets in the PPI network with a degree value of 17 or greater.

Rank	Uniport	Gene_id	Gene symbol	Definition	Degree
1	P01584	3553	IL1B	Interleukin 1 beta	48
2	P00533	1956	EGFR	Epidermal growth factor receptor	46
3	P42574	836	CASP3	Caspase 3	44
4	P03372	2099	ESR1	Estrogen receptor 1	41
5	P35354	5743	PTGS2	Prostaglandin‐endoperoxide synthase 2	39
6	P05412	3725	JUN	Jun proto‐oncogene, AP‐1 transcription factor subunit	38
7	P12931	6714	SRC	SRC proto‐oncogene, non‐receptor tyrosine kinase	33
8	P37231	5468	PPARG	Peroxisome proliferator activated receptor gamma	33
9	P45983	5599	MAPK8	Mitogen‐activated protein kinase 8	26
10	P10275	367	AR	Androgen receptor	24
11	P24941	1017	CDK2	Cyclin dependent kinase 2	24
12	P06401	5241	PGR	Progesterone receptor	23
13	Q16539	1432	MAPK14	Mitogen‐activated protein kinase 14	23
14	P17252	5578	PRKCA	Protein kinase C alpha	20
15	P08069	3480	IGF1R	Insulin like growth factor 1 receptor	20
16	P20248	890	CCNA2	Cyclin A2	20
17	P11511	1588	CYP19A1	Cytochrome P450 family 19 subfamily A member 1	19
18	Q92731	2100	ESR2	Estrogen receptor 2	19
19	P00374	1719	DHFR	Dihydrofolate reductase	18
20	P11142	3312	HSPA8	Heat shock protein family A (Hsp70) member 8	18
21	P29474	4846	NOS3	Nitric oxide synthase 3	18
22	P04035	3156	HMGCR	3‐hydroxy‐3‐methylglutaryl‐CoA reductase	17
23	O14757	1111	CHEK1	Checkpoint kinase 1	17

### 3.5 Validation of the Three Core Targets Using GEO'S Dataset

**FIGURE 6:**
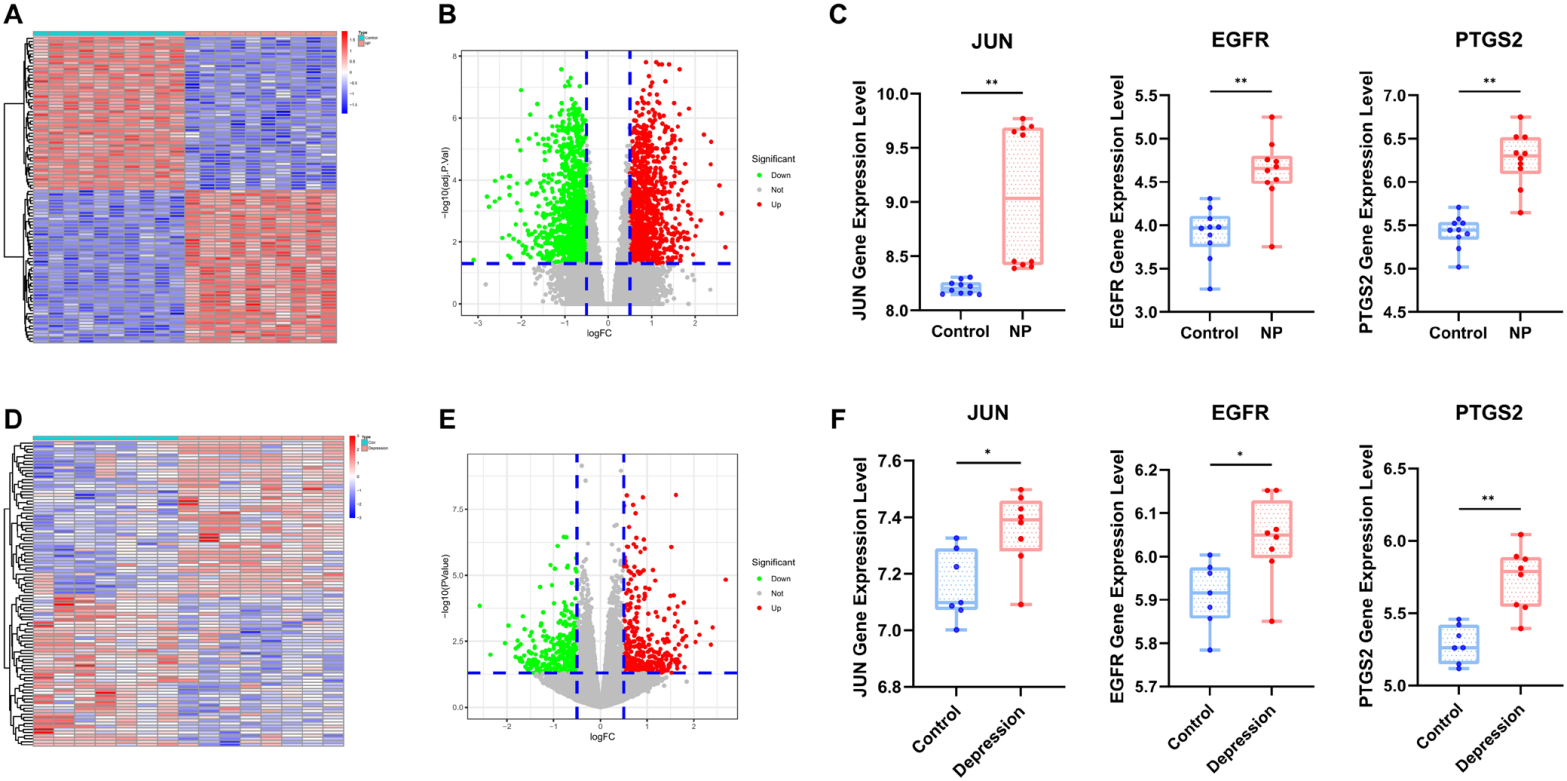
Validation of core targets in neuropathic pain and depression datasets from GEO. (A, D) The expression of DEGs in the heatmap. (B, E) The volcano map of DEGs analysis. (C, F) Three hub genes, JUN, EGFR, and PTGS2 levels from the GEO datasets used in this investigation, respectively (Con VS NP/Depression, *p < 0.05, **p < 0.01).

We utilized three datasets (GSE24982, GSE81672, and GSE146845) from the GEO database for differential expression analysis. After initial data preprocessing, including the removal of batch effects, the “limma” R package was used to screen for potential target genes. The number of differentially expressed genes (DEGs) was visualized using volcano plots (Figure 6B,E), where red dots represent upregulated genes and green dots represent downregulated genes. Meanwhile, the expression distribution of DEGs was further demonstrated by a heat map (Figure 6A,D). The analysis showed that the three core targets JUN, EGFR, and PTGS2 were all up‐regulated in the neuropathic pain and depression dataset (Figure 6C,F).

### 3.6 MD Simulation

**FIGURE 7:**
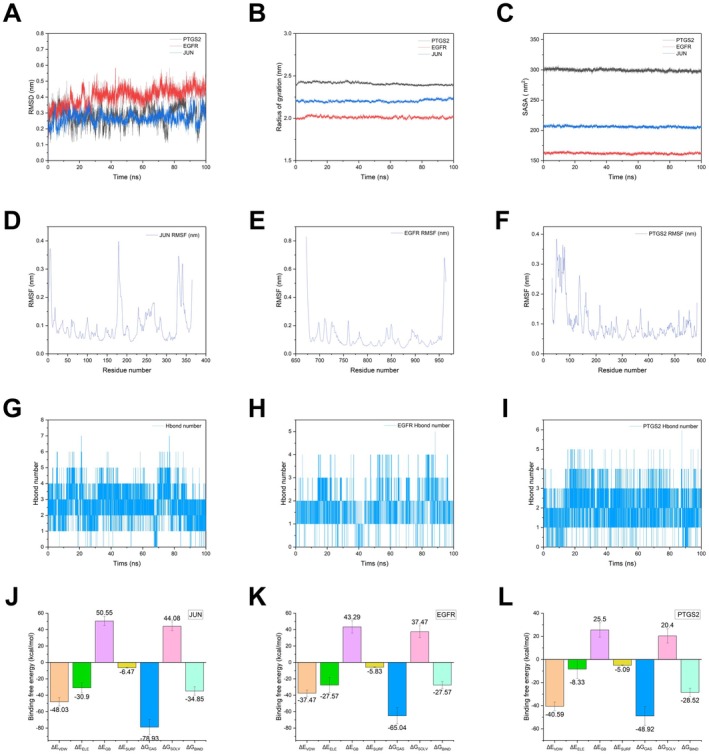
Schematic representation of MD simulation results of EB and three core targets. (A) Complex root mean square deviation (RMSD) difference over time. (B) Analysis of protein folding state and overall conformation. (C) Analysis of solvent accessible surface area (SASA). (D–F) The changes in the stability of protein targets (JUN, EGFR, PTGS2) at the residue level. (G–I) The changes in the number of hydrogen bonds between small molecule ligands and protein receptors (JUN, EGFR, PTGS2) in complex system simulations. (J–L) The resulting plot of binding free energy between EB and core target protein (JUN, EGFR, PTGS2) MD simulation protein and ligand.

To investigate the binding stability between EB and the three core target proteins and the flexibility of the protein structure, we performed 100 ns molecular dynamics simulations using AMBER 20 software. The conformational changes of the protein backbone during the simulation were assessed by analyzing the root mean square deviation (RMSD) of the complexes. As shown in Figure 7A, the conformational changes of the three proteins after binding to EB were small, and the RMSD curves were stable without obvious mutations, indicating that the complexes were structurally stable and the compounds were not dissociated from the binding sites. The flexibility characteristics of the protein residues were further evaluated by calculating the root mean square fluctuation (RMSF) values. The results showed that the RMSF of most residues was less than 0.3 nm, and the regions with large fluctuations were mainly concentrated in the terminal or flexible loops, which did not affect the overall stability (Figure 7D–F). Figure 7B shows that the radius of gyration (RoG) of the complexes was basically constant throughout the simulation, indicating that the complexes were compact and did not undergo significant unfolding, which further verified the conformational stability of the system. In addition, the solvent accessible surface area (SASA) analysis shown in Figure 7C revealed that the complex did not undergo significant contraction or exposure during the simulation, indicating that its interaction with the solvent remained stable, further supporting its structural stability. To assess the specific protein‐ligand interactions, changes in the number of hydrogen bonds during the simulations were analyzed. The results show that each complex maintains a certain number of hydrogen bonds, which contribute to the enhancement of electrostatic interactions and binding stability (Figure 7G–I). The binding free energy analysis showed that the binding free energy of JUN with EB was −34.85 kcal/mol, which was significantly lower than that of the other proteins, indicating a stronger binding ability (Figure 7J–L). The energy decomposition showed that van der Waals forces dominated between JUN and the ligand, compared with electrostatic interactions, suggesting that the binding stability was derived from non‐covalent forces dominated by hydrophobic interactions. The interaction model of the protein‐small molecule complex at −100 ns in the molecular dynamics simulation is shown in Figure S1.

### 3.7 SNI‐Induced Hyperalgesia and Depression‐Like Behavior

**FIGURE 8:**
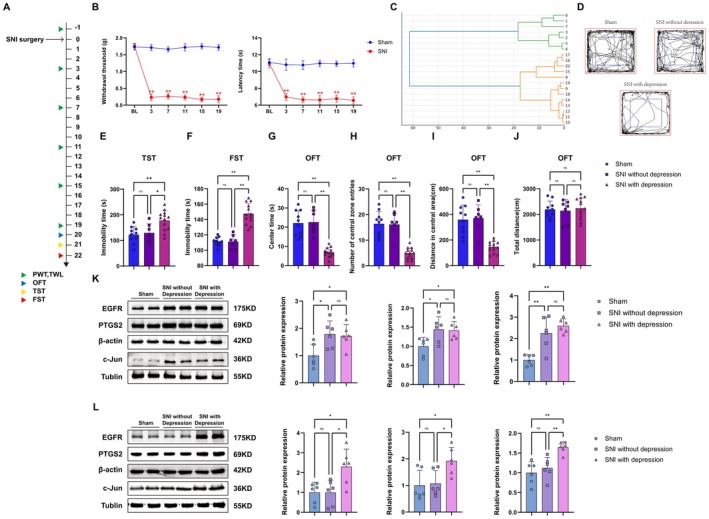
SNI‐induced hyperalgesia and depression‐like behavior. (A) Schedule of behavioral tests. (B) The mechanical withdrawal threshold and thermal withdrawal latency of the Shamand SNI mice (*n* = 20) (**p < 0.01 compared with SNI group, *n* = 10 for Sham group, *n* = 20 for SNI group). (C) SNI mice were statistically divided into two clusters by hierarchical cluster analysis. Cluster 1 (*n* = 13) was considered to be “mice with a depressive‐like phenotype,” while cluster 2 (*n* = 7) was considered to be “mice without a depressive‐like phenotype,” (D) The trajectory map in the OFT, the blue line, represents the central area. (E, F) The immobility duration during the TST and FST recording time. (G) Residence time in the center area of mice in each group. (H) The number of mice entering the center area in each group. (I) The movement distance of the central region of mice in each group. (J) Total movement distance of mice in each group (*p < 0.05, **p < 0.01, *n* = 10 for sham group, *n* = 7 for SNI without depression group, *n* = 13 for SNI with depression). (K, L) WB analysis of three core target proteins (EGFR, PTGS2, and c‐Jun) in the spinal cord and hippocampal tissue of mice in each group (*p < 0.05, **p < 0.01, *n* = 6 for each group).

Our animal experimental process is shown in Figure 8A. Compared with sham surgery, SNI mice exhibited significant decreases in MWT and TWL on postoperative days 3, 7, 11, 15, and 19 (Figure 8B). Depression behavior tests (open field test, tail suspension test, and forced swimming test) were conducted on postoperative days 20/21/22. To distinguish between depressive and non‐depressive phenotypes, the depressive behavioral data obtained from SNI mice were subjected to hierarchical cluster analysis. The results showed that 13 out of 20 rats exhibited depressive‐like phenotypes and were classified as “mice with depressive‐like phenotypes” (depressive), while the remaining rats were classified as “mice without depressive‐like phenotypes” (non‐depressive) (Figure 8C). The tail suspension test and forced swim test results showed that SNI‐induced depressive mice exhibited prolonged immobility time (Figure 8E,F). The open field test results indicated that SNI‐induced depressive mice had significantly lower central zone movement distance, central zone dwell time, and number of entries into the central zone compared to the sham surgery group, while total movement distance showed no significant difference (Figure 8D,G–J). These results confirm that chronic neuropathic pain induces depressive‐like behavior in mice. Next, we used Western blot (WB) experiments to further validate the core target proteins identified through bioinformatics and network pharmacology screening. The expression levels of EGFR, PTGS2, and Jun proteins in the spinal cord and hippocampus of mice in each group are shown in Figure 8K,L. In spinal cord tissue, compared with the sham group, the SNI‐induced depression group exhibited significantly higher expression levels of EGFR, PTGS2, and Jun compared with the non‐depression group. In hippocampal tissue, SNI model mice also exhibited upregulation of EGFR, PTGS2, and Jun protein expression. However, there were no statistically significant differences in EGFR, PTGS2, and Jun expression in the SNI group without depression.

### 3.8 EB Alleviates Pain and Depression‐Like Behavior in SNI‐Depressed Mice

**FIGURE 9:**
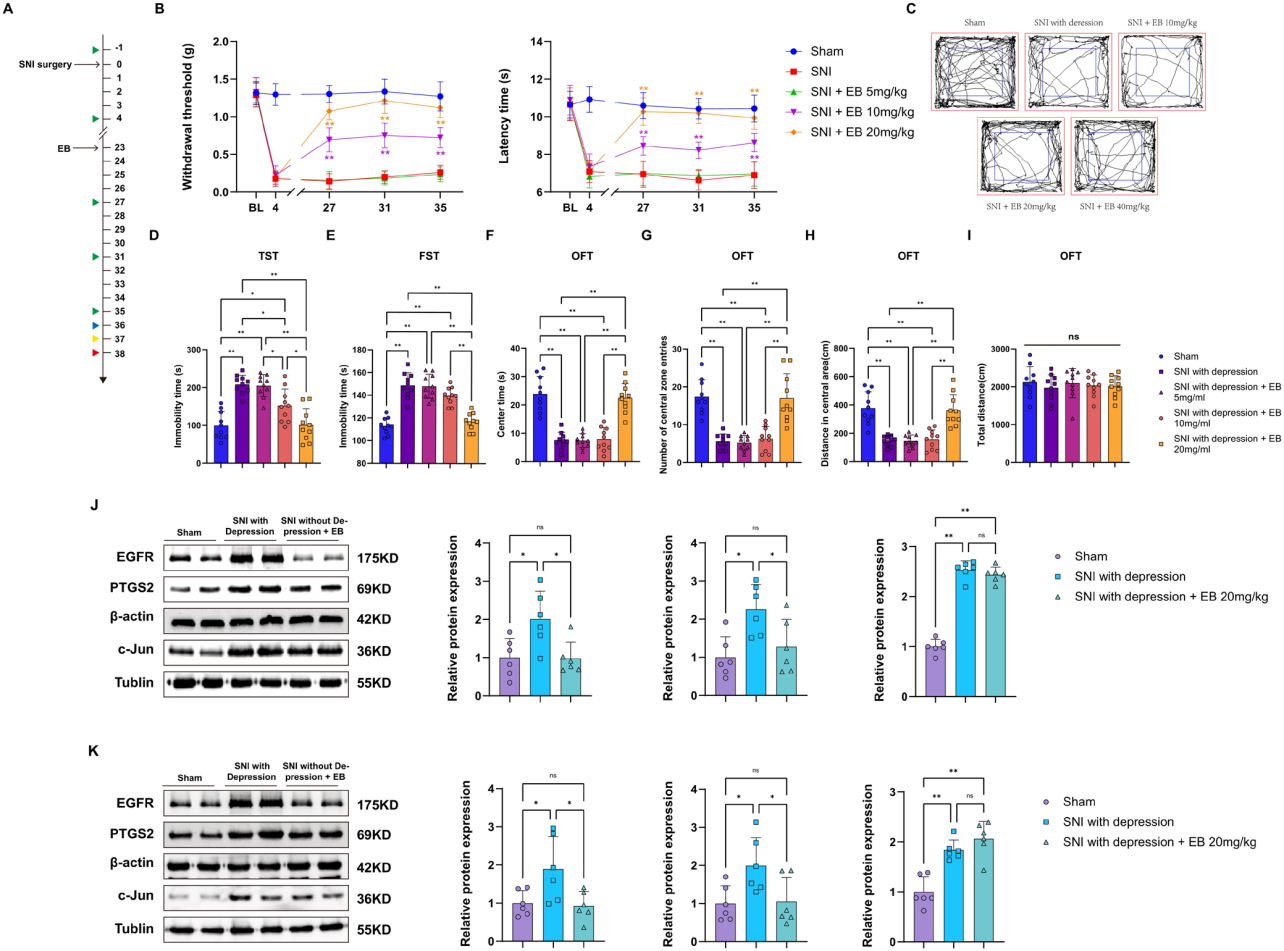
EB alleviates pain and depression‐like behavior in SNI‐depressed mice. (A) Schedule of behavioral tests. (B) The mechanical withdrawal threshold and thermal withdrawal latency of the Sham/SNI mice (**p < 0.01 compared with SNI group, *n* = 10 for Sham group, *n* = 40 for SNI group). (C) The trajectory map in the OFT, the blue line, represents the central area. (D, E) The immobility duration during the TST and FST recording time. (F) Residence time in the center area of mice in each group. (G) The number of mice entering the center area in each group. (H) The movement distance of the central region of mice in each group. (I) Total movement distance of mice in each group (*p < 0.05, **p < 0.01, *n* = 10 for each group). (J, K) WB analysis of three core target proteins (EGFR, PTGS2, and c‐Jun) in the spinal cord and hippocampal tissue of mice in each group (*p < 0.05, **p < 0.01, *n* = 6 for each group).

Over a 16‐day period, SNI mice were administered different doses of EB via intraperitoneal injection (Figure 9A). Compared to the Sham group, EB at 20 mg/kg most significantly improved mechanical hyperalgesia in SNI mice and prolonged the latency period for thermal pain (Figure 9B). In the FST and TST, EB 20 mg/kg reversed the immobility time in SNI‐depressed mice (Figure 9D,E). In the OFT, EB intervention restored the locomotor distance, central zone dwell time, and number of entries into the central zone in SNI‐depressed mice, and there were no significant differences in total locomotor distance among the groups (Figure 9C,F–I). In subsequent experiments, 20 mg/kg was determined as the optimal dose of EB and was adopted. Next, we used WB experiments to detect changes in the expression of three core target proteins after EB administration. In spinal cord tissue, the expression of EGFR and PTGS2 was significantly reduced after EB 20 mg/kg treatment, but Jun protein expression was not reduced (Figure 9J). Similar changes were observed in the hippocampus of SNI‐depressed mice (Figure 9K).

### 3.9 EB Inhibits Glial Cell Activation and Reduces Neuronal Damage

**FIGURE 10:**
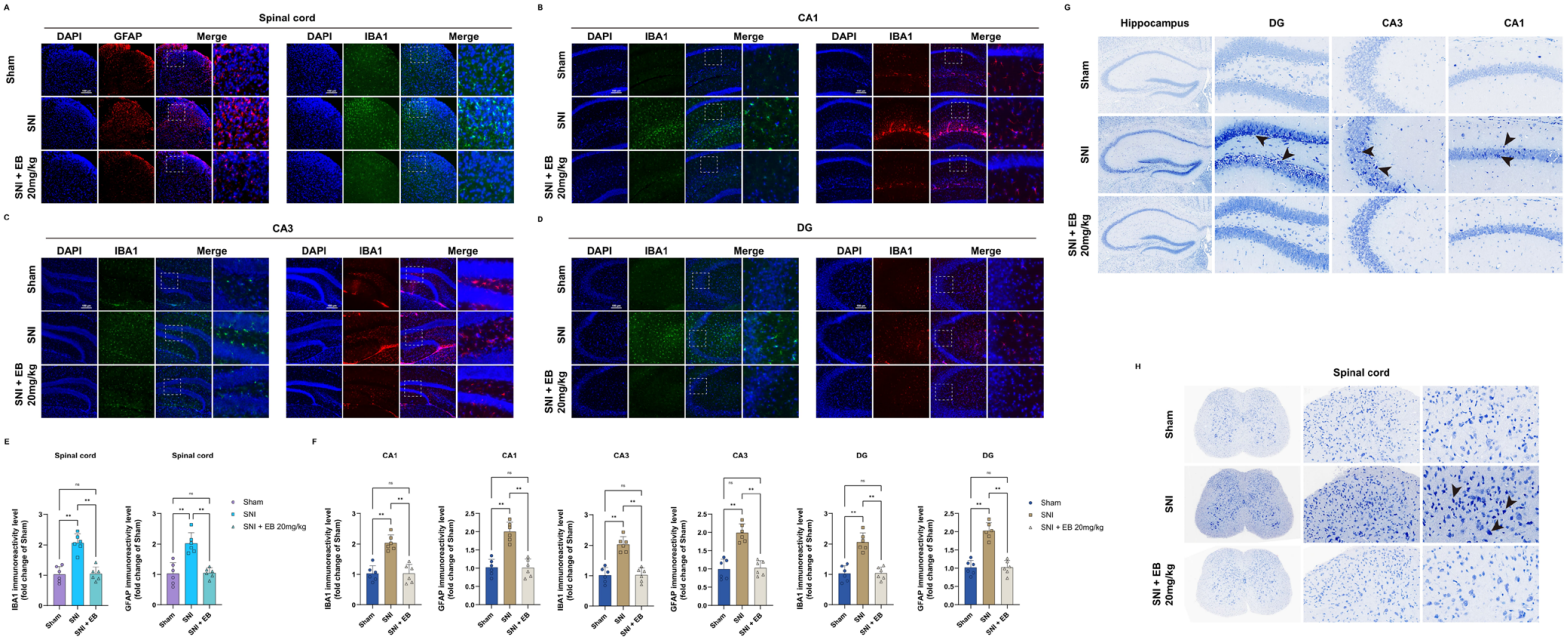
EB inhibits glial cell activation and reduces neuronal damage. (A, E) The expression of IBA1 and GFAP was detected through immunofluorescence and quantitative analysis of IBA1 in the spinal cord area, scale bar = 150 μm (**p < 0.01, *n* = 6 for each group). (B–D, F) The expression of IBA1 and GFAP was detected through immunofluorescence and quantitative analysis of IBA1 and GFAP in the hippocampus, scale bar = 150 μm (CA1, CA3, DG, **p < 0.01, *n* = 6 for each group). (G, H) Nissl staining results of spinal cord and hippocampus.

We selected the spinal cord and hippocampus for immunofluorescence analysis to explore the potential mechanisms underlying the alleviation of pain and depression co‐morbidity in SNI mice by EB. Immunofluorescence staining of the spinal cord and hippocampus revealed an increase in IBA1^+^ microglia and GFAP^+^ astrocytes in the SNI depression group mice compared to the Sham group. EB 20 mg/kg treatment reduced the activation of microglia and astrocytes in SNI‐depressed mice (Figure 10A–F). Nissl staining results showed that neurons in the spinal cord and hippocampus of SNI‐depressed mice were loosely arranged, with some cells exhibiting wrinkles and a reduced number of Nissl bodies. Treatment with EB 20 mg/kg improved this situation (Figure 10G,H).

### 3.10 EB Restored the Levels of Synaptic Plasticity‐Related Proteins in the Hippocampus of SNI‐Depressed Mice

**FIGURE 11:**
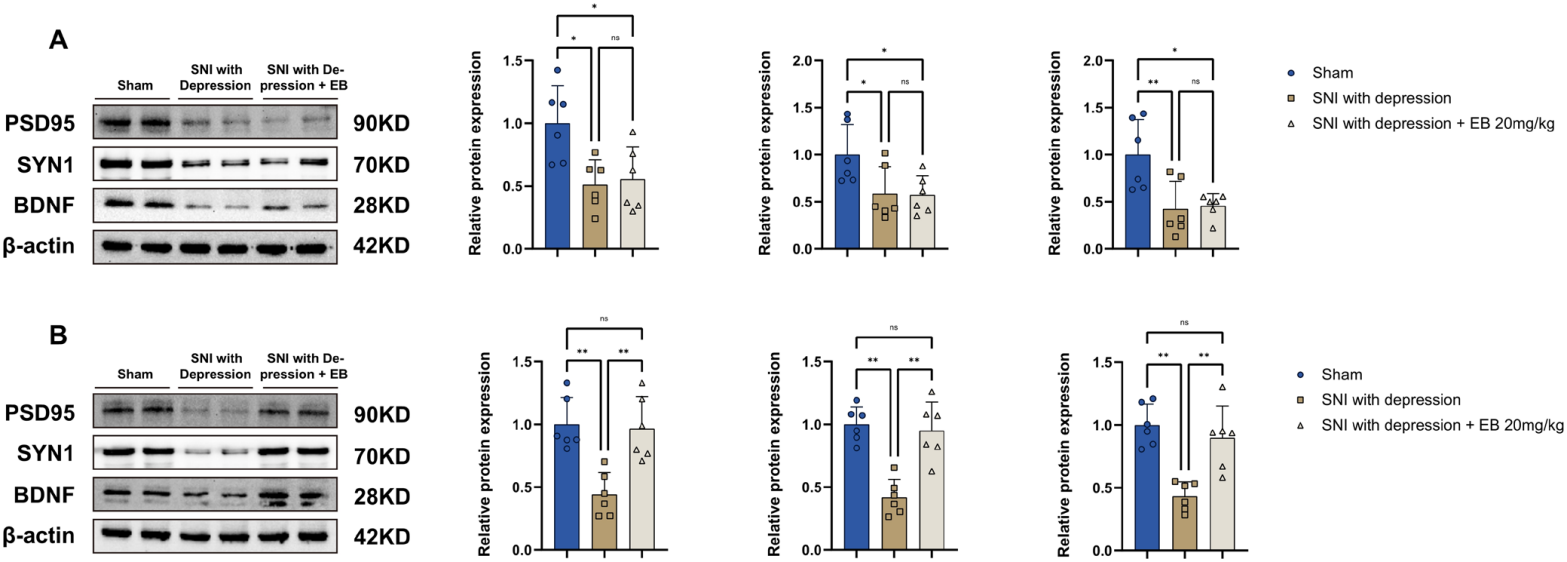
EB restored the levels of synaptic plasticity‐related proteins. (A) WB analysis of synaptic proteins including PSD95, SYN1, and BDNF in the spinal cord. (B) WB analysis of synaptic proteins including PSD95, SYN1, and BDNF in the hippocampus (*p < 0.05, **p < 0.01, *n* = 6 for each group).

Previous studies have shown that impaired synaptic plasticity is associated with neuroinflammatory neurological disorders such as depression [25] and neuropathic pain [26], so we measured the levels of synaptic plasticity‐related proteins in the spinal cord and hippocampus of SNI‐induced depressive mice. The results showed that the expression of PSD95 (a marker for postsynaptic proteins), SYN1 (a marker for presynaptic proteins), and BDNF (brain‐derived neurotrophic factor) was significantly reduced in the spinal cord and hippocampus of SNI‐induced depressive mice. Treatment with EB 20 mg/kg upregulates the expression of synaptic plasticity‐related proteins in the hippocampus, but this effect was not observed in spinal cord tissue (Figure 11A,B).

## 4 Discussion

Pain is a complex sensory experience that affects cognition, emotion, and behavior [27]. Among them, the co‐morbidity of chronic pain and depressive disorders has become an important issue in global public health. A systematic review and meta‐analysis that included 376 studies covering 347,468 chronic pain patients from 50 countries showed that approximately 39.3% of patients had clinically significant depressive symptoms and 40.2% had anxiety symptoms [28]. The occurrence of chronic pain and depression co‐morbidity is not a simple psychological response but may stem from shared neurobiological mechanisms such as impaired synaptic plasticity in the hippocampus, monoaminergic dysfunction, neuroinflammatory activation, and thalamo‐cortical–limbic loop dysfunction [29, 30, 31]. These intersecting mechanisms lead to synergistic disruptions in emotion regulation and pain perception systems, creating indistinguishable clinical presentations and increasing the complexity of diagnosis and intervention. The development of new therapeutic targets and strategies for the treatment of pain and depression is of great importance. In this study, we mainly focused on the effect of EB on SNI mice with depressive behaviors. Our research results showed that the therapeutic effect of EB might be achieved by down‐regulating EGFR and PTGS2, thereby inhibiting neuroinflammation and restoring hippocampal synaptic plasticity, ultimately improving the pain and depressive behaviors of SNI mice.

In recent years, research on natural compounds has received great attention worldwide. Among them, there have been many studies on the small molecule compound EB, such as its potential therapeutic role in allergic asthma by improving airway inflammatory markers [32]; targeting USP7 to inhibit neuroinflammation to alleviate symptoms in mouse models of dementia and Parkinson's disease [19]; and amelioration of depressive behaviors by attenuating PC12 cell damage [33]. We have taken a novel perspective, that is, the bioinformatics and cyberpharmacology approaches to understand the complex mechanisms by which the small molecule compound EB interacts with its targets. The results of the combined GO and KEGG analyses suggest that the mechanisms by which EB alleviates neuropathic pain and depression are mainly related to inflammatory responses and oxidative stress modulation. KEGG analyses showed that the mechanisms by which EB treats neuropathic pain and depression mainly include neuroactive ligand‐receptor interactions, tryptophan metabolism, inflammatory mediators of TRP channel regulatory pathways, 5‐hydroxytryptaminergic synapses and neurodegeneration. Molecular docking and MD simulations allowed us to finally identify the three most relevant targets of EB for NP and DP, including JUN, EGFR, and PTGS2. Finally, we further validated the results of the bioinformatics analyses by animal experiments.

JUN/c‐JUN is a transcription factor with a basic leucine zipper (bZIP) structure, a representative member of the activator protein‐1 (AP‐1) family, capable of binding DNA in homo‐ or heterodimeric forms to regulate target gene expression and interacting with a variety of cofactors [34]. Activation of c‐JUN is dependent on the c‐Jun N‐terminal kinase (JNK) signaling pathway, which is important in the inflammatory response, the development of neuropathic pain, and the maintenance of chronic pain. c‐Jun N‐terminal kinase (JNK) activation is involved in the development and persistence of inflammatory and neuropathic pain and the maintenance of chronic pain [35]. Huang et al. found that c‐JUN expression levels were significantly elevated in the dorsal horn region of the rat spinal cord in a chronic postoperative pain model after open‐heart surgery. Knockdown of the Ror2 gene significantly down‐regulated the expression of c‐JUN and attenuated mechanical nociceptive sensitization triggered by thoracic surgery and cold‐induced abnormal pain [36]. In addition, c‐JUN is also strongly associated with depressive symptoms, and it is involved in the regulation of several inflammation‐related factors, and inflammatory mechanisms are thought to play a central role in the pathogenesis of depression [37, 38, 39]. Recent studies have further shown that stress exposure can lead to a significant upregulation of c‐JUN expression levels in the hippocampus and prefrontal cortex of rats, which affects the onset and maintenance of depressive behaviors [40, 41]. Although the upregulation of c‐Jun is closely related to the occurrence of neuropathic pain and depression, in our study, it was found that EB treatment could significantly reduce the expression levels of its downstream functional effect molecules EGFR and PTGS2, but did not significantly decrease the increased c‐Jun protein content in the hippocampus of SNI combined with depression mice. This phenomenon does not necessarily contradict the target screening strategy, but may reflect the essential differences in the regulatory mechanisms of transcription factors and downstream effect molecules. As a transcription factor, the biological function of c‐Jun mainly depends on post‐translational modifications (especially phosphorylation activation mediated by the JNK signaling pathway), rather than simple changes in protein total quantity [42]. Therefore, EB may exert its effect by regulating the active state of c‐Jun (such as inhibiting its phosphorylation level or downstream transcriptional activity), rather than directly reducing its protein expression level. Moreover, the target screening strategy of this study is based on network topological parameters (Degree centrality), molecular docking binding energy, and GEO dataset validation, and what is identified are the “key regulatory nodes” in the disease network, rather than ensuring that all core targets show the same direction and amplitude of expression changes in vivo. In complex biological regulatory networks, drug effects often manifest as selective regulation of specific signaling pathways rather than synchronous inhibition of all network nodes. Therefore, the inclusion of JUN more reflects its hub status in the disease network, while the actual therapeutic effect of EB may be achieved indirectly by regulating the functional state of JUN‐related signaling pathways, rather than directly reducing its protein content. In the future, by detecting p‐c‐Jun and JNK signaling pathway activity, the specific role of c‐Jun in the EB treatment mechanism will be further clarified.

EGFR (epidermal growth factor receptor) is a member of the ErbB family, which consists of four different receptor tyrosine kinases [43] and can regulate pain [44, 45]. The small molecule inhibitors of EGFR (gefitinib and lapatinib) have a significant analgesic effect on inflammatory pain and neuropathic pain in mice [45]. The use of clinically available compounds to inhibit EGFR also significantly reduced pain‐related defensive behaviors in mouse models of inflammatory pain and chronic pain [46]. EGFR is also involved in emotional regulation. A 2015 genome‐wide association study found that EGFR is associated with bipolar affective disorder [47]. Additionally, the computational biology research conducted by Zhu et al. also revealed that EGFR is associated with bipolar depression [48], which is consistent with our research results—EGFR was identified as one of the key hub nodes in the network pharmacology analysis and showed a significant upregulation trend in the SNI combined with depression mouse model. More importantly, EB intervention could significantly downregulate the protein expression level of EGFR in the hippocampal tissue, accompanied by the improvement of pain, depressive‐like behaviors, and neuroinflammation. The consistency in direction between the molecular expression changes and the behavioral improvement suggests that EGFR may play an important regulatory role in the co‐occurrence of pain and depression.

PTGS2 (Cyclooxygenase‐2), also known as prostaglandin synthetase 2, regulates inflammation and homeostasis in vivo through the synthesis of lipid mediators such as prostaglandins [49], and its activation state is usually closely associated with injury and inflammation [50]. PTGS2 plays an important role in pain. High expression of PTGS2 exacerbates inflammatory neuropathic injury triggered by microglial activation, thereby inducing pain perception and inflammatory responses; specifically inhibiting PTGS2 reduces oxidative stress levels and pro‐inflammatory cytokine concentrations, thereby alleviating neuropathic pain [51]. In addition, PTGS2 is also closely related to depressive symptoms, and it has been shown that PTGS2 over‐activation may trigger neurotransmitter disorders, induce neuroinflammation, and affect neuroplasticity, thus playing an important role in the onset and persistence of depression [52]. Unlike previous studies that mainly focused on the role of PTGS2 in single pain or depression models, this study, conducted in the context of a pain‐depression comorbidity model, further verified the abnormal activation of PTGS2 in the central nervous system and its responsiveness to EB intervention. This result indicates that the improvement of EB on neuropathic pain and depressive‐like behaviors may be partially dependent on its inhibitory effect on the inflammatory cascade reaction mediated by PTGS2.

Synaptic plasticity impairment is a hallmark of many neuroinflammatory‐related neurological disorders, such as Alzheimer's disease and depression [25, 53]. Numerous studies have reported alterations in hippocampal neurogenesis in chronic pain animal models, which may underlie the associated emotional and cognitive dysfunctions. In this study, we observed a significant decrease in the expression levels of synaptic proteins (PSD95, SYN1, and BDNF) in mice exhibiting comorbid pain and depression. This aligns with previous findings that SNI disrupts hippocampal synaptic plasticity, as evidenced by long‐term potentiation (LTP) deficits and reduced excitatory synapses [26]. Morphological investigations have also demonstrated that SNI diminishes dendritic spine density and dendritic complexity in hippocampal pyramidal neurons [54]. Moreover, Liu et al. highlighted that SNI‐triggered microglial activation compromises synaptic structure and functional plasticity in the hippocampus [9]. In our study, administration of EB upregulated synaptic protein expression in the hippocampus and ameliorated deficits in synaptic plasticity.

Our study provides valuable insights into the mechanisms by which EB ameliorates depression‐neuropathic pain comorbidity in SNI mice, but some limitations remain. Firstly, although this study observed that EGFR and PTGS2 expression are regulated by EB and are accompanied by the restoration of synaptic protein (PSD95, SYN1, and BDNF) levels, these findings remain correlational evidence and cannot yet establish a direct causal relationship. Moving forward, we will conduct additional in vivo and in vitro experiments to validate potential causal mechanisms, such as gene knockout/knockdown or the use of targeted specific drug inhibitors. Secondly, this study focused on specific target genes related to pain and depression; although these genes have been involved in the relevant pathways, there may be other key genes and molecular mechanisms that have not been included and should be expanded to a wider genomic map in the future. Finally, long‐term studies are needed to assess the continued efficacy and safety of EB.

In summary, this study is the first to combine network pharmacology, molecular docking, molecular dynamics simulation, bioinformatics and experimental methods to systematically investigate the pharmacology and molecular mechanism of the small molecule compound EB for the treatment of pain and depression co‐morbidities. We identified c‐Jun, EGFR and PTGS2 as the key target genes that EB may act on. EB inhibited the activation of microglia in the spinal cord and hippocampus by modulating these targets and ameliorated synaptic plasticity impairments by up‐regulating the synaptic proteins in the hippocampus, which significantly alleviated the pain and depression‐like behaviors in SNI mice.

## Author Contributions

X.Y., H.X., and K.C. designed and drafted the manuscript. X.Y. and F.J. acquired data. Y.W. analyzed the data. All authors approved the final version of the manuscript for publication.

## Funding

This work was supported by the Hospital Discipline Capacity Building Project from the Department of Finance in Hubei Province (SCZ2025014) and Hainan Provincial Natural Science Foundation of China (825MS198).

## Disclosure

All procedures were conducted in accordance with institutional ethical guidelines and approved by the Institutional Animal Care and Use Committee (IACUC) of Hubei University of Medicine (No. 2025104).

## Conflicts of Interest

The authors declare no conflicts of interest.

## Supporting information


**Figure S1:** The interaction model of EB and core target protein MD simulates 100 ns. (A) c‐Jun. (B) EGFR. (C) PTGS2.

## Data Availability

The data that support the findings of this study are available from the corresponding author upon reasonable request.
