# Supplementary material for: The Small Molecule Compound Eupalinolide B Ameliorates Depressive Behaviors and Neuropathic Pain in Mice With Spared Nerve Injury: Integrating Network Pharmacology, Molecular Docking, Bioinformatics, Molecular Dynamics Simulation and Experimental Verification
Source: CNS Neurosci Ther. 2026 Apr 10;32(4):e70872. doi: 10.1002/cns.70872 (PMC13067920; doi:10.1002/cns.70872)
Supplement: Supplementary file 1 — Figure S1: The interaction model of EB and core target protein MD simulates 100 ns. (A) c‐Jun. (B) EGFR. (C) PTGS2. [file CNS-32-e70872-s001.docx]

**Supplementary Materials and Methods**

**Neuropathic pain models**

Mice were deeply anesthetized with isoflurane and fixed on the surgical table. The sciatic nerve on one side and its three branches—the common peroneal, tibial, and sural nerves—were carefully exposed under a surgical microscope. The common peroneal and tibial nerves were tightly ligated with silk sutures and then transected, with approximately 2 mm of the distal segment removed, while the sural nerve was left intact. In the sham group, the sciatic nerve was similarly exposed but not ligated or transected.

**Behavioral experiments**

1. MWT and TWL

Mechanical sensitivity was evaluated using the von Frey test to determine MWT (mechanical withdrawal thresholds). Mice were placed individually in Plexiglas chambers (8 × 8 × 6 cm) positioned on a wire mesh floor that allowed access to the plantar surface of the paws. Animals were habituated for at least 1 h before testing. Calibrated von Frey monofilaments (Stoelting Touch Test) were applied to the plantar surface of the hind paw for 3 s, and responses were assessed following Dixon’s up-and-down paradigm to calculate the 50% withdrawal threshold[1].

Thermal sensitivity was assessed by measuring TWL (thermal withdrawal latency). Mice were placed in Plexiglas compartments of the same size on a glass plate and allowed to acclimate for at least 1 h. A focused radiant heat source (BME-410C automated plantar analgesia system; Tianjin, China) was applied to the plantar surface of the hind paw, and the latency to paw withdrawal was recorded. Each mouse was tested three times at intervals of no less than 30 min, and the mean latency was calculated. To prevent tissue damage, a cutoff time of 30 s was imposed[2].

1. TST

The TST (tail suspension test) is a widely recognized test method that assesses depressive-like behavior by measuring immobility time. Mice are suspended from a lever at the edge of a table (35 cm above the table surface), with the tip of their tails fixed to the table surface with tape at a distance of approximately 1–2 cm. Under these conditions, mice exhibit escape-oriented behavior interspersed with temporary increases in immobility episodes. The mouse's immobility time is recorded for 6 minutes[3].

1. FST

The FST (forced swimming test) is also used to evaluate depressive-like behavior in mice. During the experiment, mice are placed individually in a cylindrical container with a diameter of 15 cm, a height of 25 cm, and filled with water at 27°C, and are required to swim continuously for 6 minutes. The mice's behavior is recorded by a camera placed outside the tank and analyzed by an experimenter who is unaware of the treatment conditions. The duration of immobility during the final 4 minutes is statistically tested, with the criterion being the minimum movement required for the animal to maintain floating or balance, rather than active swimming[4].

1. OFT

Locomotor activity was evaluated using the OFT (open field test) as previously described[5, 6]. Mice were individually placed in a square arena (50 × 50 × 40 cm) and allowed to freely explore for 5 min, starting from the center of the apparatus. A video camera positioned 100–120 cm above the arena recorded the activity, which was subsequently analyzed with an automated tracking system. To eliminate residual olfactory cues, the arena was thoroughly cleaned with 75% ethanol between trials. All behavioral assessments were conducted during the light phase, between 10:00 a.m. and 6:00 p.m. The relevant videos were analyzed using the smart software (Panlab, Spain).

**Western blotting**

L4–L5 spinal cord and hippocampal tissues were collected and homogenized in RIPA lysis buffer containing PMSF. The homogenates were centrifuged at 12,000 rpm for 15 min at 4°C, and the supernatants were collected for protein quantification using a BCA assay kit. Protein samples were mixed with loading buffer and denatured at 95°C for 10 min. Equal amounts of protein were loaded onto 10% SDS–polyacrylamide gels for electrophoretic separation, followed by transfer to PVDF membranes. Membranes were blocked with 5% nonfat milk at room temperature for 2 h, and then incubated overnight at 4°C with the following primary antibodies: Rabbit anti-EGFR antibody ([1:1000], ab52894, abcam), Rabbit anti-PTGS2 antibody ([1:1000], ET1610-23, HUABIO), Rabbit anti-c-Jun antibody ([1:1000], ET1608-3, HUABIO), Rabbit anti-PSD95 antibody ([1:1000], A0131, Abclonal), Rabbit anti-SYN1([1：1000], A17362, Abclonal), and Rabbit anti-BDNF([1：1000], A11028, Abclonal). After washing with TBST, membranes were incubated with horseradish peroxidase-conjugated secondary antibodies at room temperature for 2 h. Protein signals were detected using an enhanced chemiluminescence (ECL) kit (Thermo Scientific, USA) and imaged with the ChemiDoc MP system. Band intensities were quantified using ImageJ software, and protein expression levels were normalized to β-actin. Results are presented as fold changes relative to the control group, which was set to a value of 1.

**Nissl staining and immunofluorescence staining**

Mice were deeply anesthetized with pentobarbital sodium, followed by cardiac perfusion with 20 mL of phosphate-buffered saline (PBS). Subsequently, perfusion was performed using 4% paraformaldehyde. Brain tissue and the L4-L5 spinal cord segment were collected and fixed overnight in 4% paraformaldehyde. For Nissl staining, after 48 hours of tissue fixation, dehydrate the tissue and embed it in paraffin. Section the tissue into 10-micron slices and stain with Nissl stain. For immunofluorescence detection, dehydrate the tissue in 30% sucrose, then embed it and section into 30-micron continuous slices. The sections were blocked at room temperature for 2 hours in a solution containing 5% donkey serum and 0.1% Triton X-100, then incubated at 4°C with the following primary antibodies: anti-IBA1 (1:300, ab289874, Abcam) and anti-GFAP (1:500, 173308, SYSY). After two 10-min washes with PBS, sections were incubated for 2 h at room temperature with corresponding secondary antibodies: donkey anti-goat Alexa Fluor 488 (1:400, ab150129, Abcam) and donkey anti-guinea pig Alexa Fluor 488 (1:400, D-GP488, Oasis Biofarm). Nuclei were counterstained with DAPI (Beyotime, C1005) before imaging with a fluorescence microscope (BIO-RAD, USA).

**Hierarchical cluster analysis**

Literature indicates that not all chronic pain animal models consistently exhibit depressive phenotypes[7]. A simple comparison between the “Sham group” and the “SNI group” may dilute or obscure therapeutic effects targeting the “pain-depression comorbidity” subgroup. To objectively and unbiasedly identify the true subgroup of SNI mice developing depressive-like behaviors, we employed hierarchical cluster analysis. This data-driven approach segmented SNI mice based on their combined performance across multiple depressive behavioral indicators, precisely distinguishing “mice exhibiting depressive-like phenotypes” from “mice not exhibiting depressive-like phenotypes.” Variables included in the clustering analysis comprised immobility time in the tail suspension test (TST), immobility time in the forced swim test (FST), and central zone residence time, number of entries, and locomotor distance in the open field test (OFT). Given the differing units and variability among these measures, we first Z-score normalized the raw data from all SNI mice (n=20) to eliminate unit effects and ensure equal weighting for each indicator in the clustering analysis. Hierarchical clustering was then performed using Ward's method combined with squared Euclidean distance as the metric, with results visualized as a dendrogram.


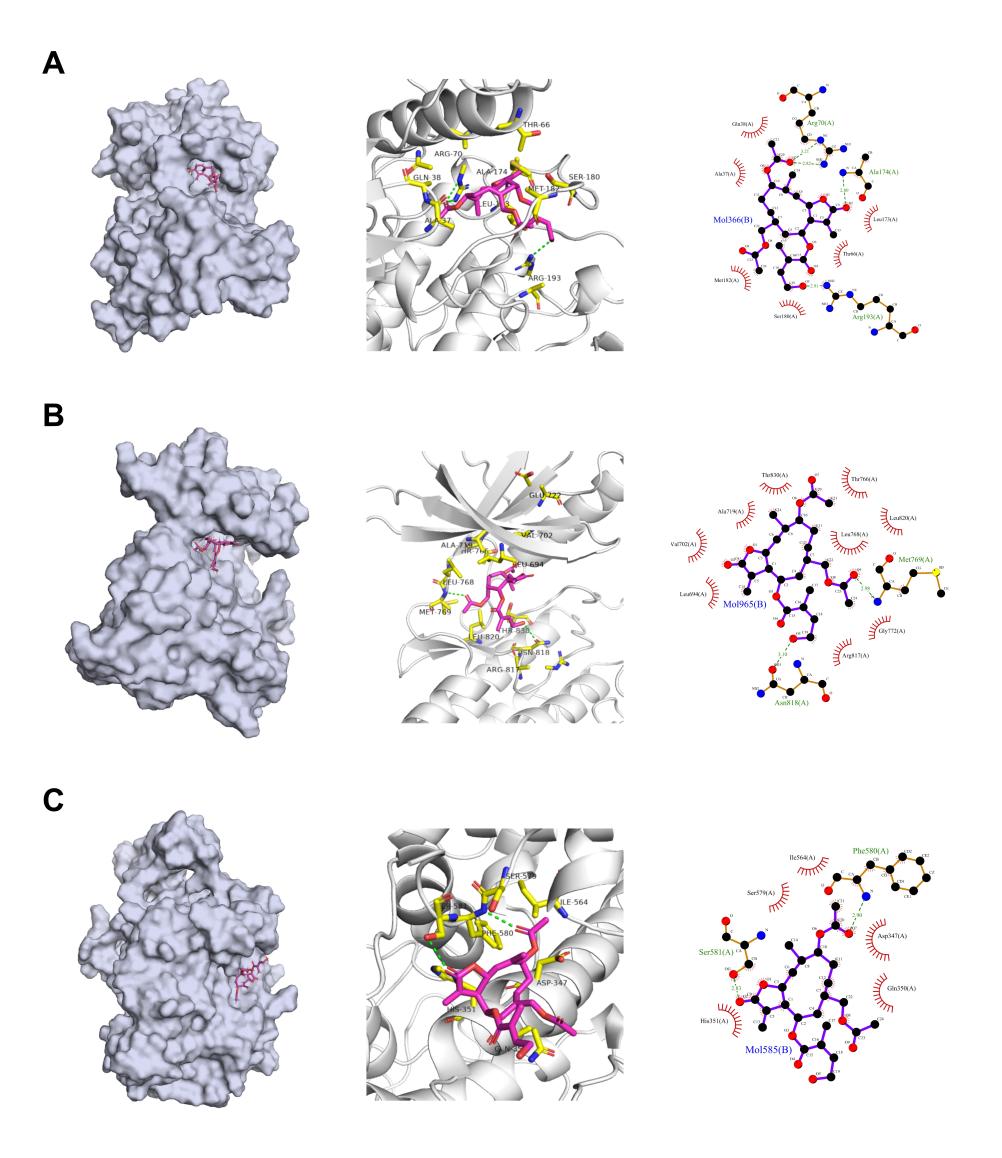


**Figure S1. The interaction model of EB and core target protein MD simulates 100 ns.** (A) c-Jun. (B) EGFR. (C) PTGS2.

## References

1. Chaplan, S.R., et al., *Quantitative assessment of tactile allodynia in the rat paw.* J Neurosci Methods, 1994. **53**(1): p. 55-63.

2. Deng, Z., et al., *Catestatin is involved in neuropathic pain mediated by purinergic receptor P2X(4) in the spinal microglia of rats.* Brain Res Bull, 2018. **142**: p. 138-146.

3. Can, A., et al., *The tail suspension test.* J Vis Exp, 2012(59): p. e3769.

4. Powell, T.R., C. Fernandes, and L.C. Schalkwyk, *Depression-Related Behavioral Tests.* Curr Protoc Mouse Biol, 2012. **2**(2): p. 119-27.

5. Zhang, T.T., et al., *Ammoxetine attenuates diabetic neuropathic pain through inhibiting microglial activation and neuroinflammation in the spinal cord.* J Neuroinflammation, 2018. **15**(1): p. 176.

6. De Gregorio, D., et al., *Cannabidiol modulates serotonergic transmission and reverses both allodynia and anxiety-like behavior in a model of neuropathic pain.* Pain, 2019. **160**(1): p. 136-150.

7. Xie, Z.M., et al., *Alterations in the inflammatory cytokines and brain-derived neurotrophic factor contribute to depression-like phenotype after spared nerve injury: improvement by ketamine.* Sci Rep, 2017. **7**(1): p. 3124.
